# Supplementary material for: A Cohort of Patients with COVID-19 in a Major Teaching Hospital in Europe
Source: J Clin Med. 2020 Jun 4;9(6):1733. doi: 10.3390/jcm9061733 (PMC7356883; doi:10.3390/jcm9061733)
Supplement: Supplementary file 1 [file jcm-09-01733-s001.pdf]

SUPLEMENTARY APPENDIX

# A cohort of patients with COVID-19 in a major teaching hospital in Europe

Table of Contents

1. COVID@HULP Working Group list of members and Aknowledgments
2. Electronic case record form (eCRF)

COVID@HULP Working Group list of members

**Conception and design of COVID@HULP**: E Ramírez, J Frías, AJ Carcas, AM Borobia, J Montserrat, JR Arribas

**Scientific Committee:** J Frías, AJ Carcas, E Ramírez, A Martín-Quirós, M Quintana, J Mingorance, F Arnalich, F Moreno, JC Figueiras, N García-Arenzana, JR Arribas, AM Borobia

**Data Collection**

Microbiology Department: M Dolores Montero, MP Romero, C Toro-Rueda, S García-Bujalance, G Ruiz-Carrascoso, E Cendejas-Bueno, I Falces-Romero, F Lázaro-Perona, M Ruiz-Bastián, A Gutiérrez-Arroyo, P Girón De Velasco-Sada, E Dahdouh, B Gómez-Arroyo, C García-Sánchez, V Guedez-López, I Bloise, M Alguacil-Guillén, M Gracia Liras-Hernández, M Sánchez-Castellano, P García-Clemente, P González-Donapetry, S San José-Villar, M de Pablos, R Gómez-Gil, M Corcuera, A. Rico-Nieto, B. Loeches, J Mingorance, J García Rodríguez

Pharmacy Department: F Moreno, A Herrero

Laboratory Medicine Department: D Prieto Arribas, P Oliver-Saez, R Mora, P Fernández-Calle, MJ Alcaide, J Diaz-Garzón, B Fernández-Puntero, R Nuñez, G Crespo, O Rodriguez, H Mendez, M Duque, R Gomez, M Sanz de Pedro, L Pascual, M Segovia, JM Iturzaeta, M Rodriguez, A García, MA Martinez, B Fabre, E Martinez, I Moreno, N Rodriguez, D Ortiz, M Simon, IG Tomoiu, C Pizarro, B Montero, AL Qasem, M Gomez, I Casares, A Buño.

Radiology Department: M Martí de Gracia, L Parra Gordo, A Diez Tascón, S Ossaba Vélez, I Pinilla, E Cuesta, M Fernández- Velilla, M Torres, G Garzón

Preventive Medicine Department: V Pérez, A Quintás, I San Juan, J Cantero, C Pérez, M Castro, L Hernández, T Pedraz, E Fernández, C García, A Robustillo.

**Clinical Team**

Emergency Department: I Fernández, M Noguerol, A Martínez, M González, R Cabrera, R Mayayo, R Marín, V Lo-Iacono, M Lerín, P Romero, B Reche, R Tejada, M Rico, R Deza, S Fabra, I Arroyo, L Dani, L Labajo, R Soriano, L López, E Calvin, S Martñinez, L López-Tappero, M Pilares, O González, G Bejarano, A Iglesias, Y Tung,

C Maroun, R Bravo, M Silvestre, F Perdomo, B Alonso, B Antón, I Arenas, C Cabré, F Marqués, E Muñoz, MA Molina, N Cancelliere, S Pastor, L Frade, P López, I García

Internal Medicine Department: F Arnalich, C Fernández Capitán, JJ González Garcia, JM Herrero, MA Quesada Simón, A Robles Marhuenda, JI Bernardino, M Mora, C Soto Abanedes, AM Noblejas Mozo, JC Ramos, B Diaz Pollán, MJ Jaras Hernandez, E Martinez Robles, A Moreno Fernandez, R Montejano, A Sanchez Purificación, JC Martin Gutiérrez, PL Martinez Hernández, F la Calle, M Arsuaga, M Diaz Menéndez, E Trigo, C Busca Arenzana, T Sancho Bueso, A Lorenzo Hernández, B Gutierrez Sancerni, G. Salgueiro, L. Martin Carbonero, J. Mostaza, R. de Miguel, M.A. Martinez López, V. Hontañon, A. Menéndez, J. Cadiñanos, J Alvarez Troncoso, A Castellano, C Marcelo Calvo, I Vives Beltrán, L Ramos Ruperto, G Daroca Bengoa, MM Arcos Rueda, J Vasquez Manau, P Fernández Cidón, C Rosario Herrero Gil, E Palmier Peláez, Y Untoria Tabares, C. Lahoz, E. Estirado, C Hernández, F. Garcia-Iglesias, E. Monteoliva, M Martínez, M. Varas, T. González Alegre, ME Valencia, V Moreno, MLMontes

Neumology Department: S Alcolea, J Cabanillas, C Carpio, R Casitas, J Fernández Bujarrabal, I Fernández Navarro, J Fernández Lahera, C García Quero, M Hidalgo, R Galera, F García Río, L Gómez Carrera, M Gómez Mendieta, A Mangas, E Martínez Cerón, M Martínez Redondo, Y Martínez Abad, A Martínez Verdasco, C Plaza, Sarai Quirós, D Romera, D Romero, B Sánchez, A Santiago, C Villasante, E Zamarrón, V Arnalich, P Mariscal, A Falcone, D Laorden, MC Prados, R Alvarez Sala

Intensive Care Department: A García, C Arévalo, C Gutiérrez, JC Figueira, M Quintana, S Yus, MJ Asensio, M Sánchez, JM Añón, J Manzanares, A Garcí de Lorenzo, E Perales, B Civantos, L Cachafeiro, A Agrifoglio, B Estébanez, E Flores, M Hernández, P Millán, M Rodríguez, C Gutiérrez, K Nanwani

Pediatric Intensive Care Department: B Arizcun, E Pérez, D Rodríguez, M Sánchez, U Quesada, C Román, P Dorao, E Alvarez-Rojas, JJ Menendez-Suso, C Verdu, A Gómez-Zamora, C Schuffelman, B Calderón, M Laplaza, M del Rio, I Amores, M Rodriguez-Rubio, P de la Oliva

Cardiology Department: J Ruiz, S Rosillo, O González, A Iniesta, I Ponz.

Anesthesiology Department: JM Muñoz Ramón, MC Hernández Gancedo, R Uña Orejón, P Sanabria Carretero, I Moreno Gomez-Limón, A Seiz Martinez, E Guasch Arévalo, C Martín-Carrasco, E Alvar, L Serrá, F Iannucelli, J Latorre, S Casares, I Valbuena, L Diaz Díez Picazo, C Rodríguez Roca: O Cervera, E García de las Heras, P Durán, C Castro, C Manrique de Lara, J Veganzones, A López Tofiño, E Fernandez Cerezo, S Zurita, S Casares, M Lopez Martinez, T Prim, J Alvárez del Vayo, G Alcaraz,L Castro, J Yagüe, S Díaz-Carrasco, P González-Pizarro, A Montero, FJ Sagra, A Suárez

**Data Entry:**

Medical Students: F Abellán, J Alonso, A Álvarez, M Archinà, S Arribas, T Baselga, P Barco, N Barrera, L Barrera, A Bartrina, G Bassani, P Betancort, I Blanco, C Blasco, L Brieba, F Cadenas, P Carrera, C Cascajares, A Catino, R Cavallé, D Ceniza, Y Conde, L Currás, M Daltro, A Esteban, M Fernández, I Ferrer, L Regaño, P Galindo, S Garcia-Bellido, C García-Mochales, T Gómez, C Gómez, N González, S González, J Guisández, P Hernández, R Hernando, I Llorente, A Marín, P López, L Mejuto, M Palma, A Peña, L Platero, D Pujol, M Ramírez, M Redondo, F Reinoso, A Rodríguez, A Rodríguez, L Romero, S Sánchez, M Sánchez, P Serrano, H Serrano, T Silva, E Soria, A Suárez, B Tejero, A Torrecillas, J Torres, M Valentín-Pastrana, A Villanueva, M Virgós, M Yagüe, N Yustas.

Clinical Pharmacology Department: J Montserrat, J Queiruga, A Rodriguez Mariblanca, L Martínez de Soto, M Urroz, E Seco, M Zubimendi, S Stuart, L Díaz, I García

**Data management:**

Scientific Support Unit. Health Research Institute "Hospital 12 de Octubre": MT García Morales

Clinical Data management. La Paz University Hospital: A Martín-Vega

Monitoring: Clinical Trial Unit (UCICEC). Clinical Pharmacology Department.

**Data revision and analysis**: J Montserrat, A Caro, AM Borobia, AJ Carcas, J Frías, E Ramírez, G Martínez-Alés, JR Arribas

Acknowledgments

We thank all La Paz Hospital staff, whose selfless dedication is allowing us to face the worst healthcare challenge of our history. We also want to acknowledge Hospital Management for its support to the Covid@HULP project.

Electronic case record form (eCRF)

Unidad de Investigación y Soporte Científico

## HULP-COVID19-ACE2-20

Project Home | Project Setup | Codebook

# Data Dictionary Codebook

03/04/2020 12:08

| # | Variable / Field Name | Field Label<br>*Field Note* | Field Attributes (Field Type, Validation, Choices, Calculations, etc.) |
|---|---|---|---|
| Instrument: **Datos Demograficos** (datos_demograficos) | | | |
| 1 | record_id | Record ID | text<br>Custom alignment: RH |
| 2 | nhc | NHC | text |
| 3 | cipa | CIPA | text |
| 4 | sexo | Sexo al nacer | radio<br>1 Hombre<br>2 Mujer<br>Custom alignment: RH |
| 5 | edad | Edad<br>*años* | text (integer, Min: 0, Max: 120) |
| 6 | codigo_postal | Codigo Postal | text (zipcode)<br>Custom alignment: RH |
| 7 | condiciones_acinamiento | Section Header:<br>Condiciones de acinamiento | radio<br>0 No<br>1 Residencia<br>2 Albergue<br>3 Centro penitenciario<br>Custom alignment: LH |
| 8 | convivencia_covid_positivo | Convivencia con un COVID POSITIVO | radio<br>0 No<br>1 Si<br>Custom alignment: RH |
| 9 | pais_nacimiento | Pais de nacimiento | text<br>Custom alignment: RH |
| 10 | estadio_funcional | Estadio funcional | radio<br>1 Dependiente para Actividades Básicas de la Vida Diaria<br>2 Semidependiente para Actividades Básicas de la Vida Diaria<br>3 Independiente para Actividades Básicas de la Vida Diaria<br>Custom alignment: LV |
| 11 | sospecha_transmision_nosocomial | Sospecha de Transmisión Nosocomial | radio<br>0 No<br>1 Si<br>Custom alignment: RH |
| 12 | centro_ingreso | Section Header:<br>Centro de Ingreso | text<br>Custom alignment: RH |
| 13 | habitacion | Habitacion | text<br>Custom alignment: RH |
| 14 | planta_ingreso | Planta Ingreso | text<br>Custom alignment: RH |

| | | | |
|---|---|---|---|
| 15 | reingreso | Reingreso | radio<br>0 No<br>1 Si<br><br>Custom alignment: RH |
| 16 | datos_demograficos_complete | Section Header: *Form Status*<br>Complete? | dropdown<br>0 Incomplete<br>1 Unverified<br>2 Complete |
| Instrument: **Antecedentes Personales** (antecedentes_personales) | | | |
| 17 | enf_cardiacas_cronicas | Enfermedades cardíacas crónicas, incluyendo enfermedades cardíacas congénitas (no hipertensión) | radio<br>0 No<br>1 Si<br><br>Custom alignment: RH |
| 18 | hipertension | Hipertensión | radio<br>0 No<br>1 Si<br><br>Custom alignment: RH |
| 19 | enf_pulmonar_cronica | Enfermedad pulmonar cronica | radio<br>0 No<br>1 Si<br><br>Custom alignment: RH |
| 20 | epoc | EPOC (no asma) | radio<br>0 No<br>1 Si<br><br>Custom alignment: RH |
| 21 | necesidad_o2_domiciliario<br>Show the field ONLY if:<br>[epoc] = '1' | Necesidad de O2 domiciliario | radio<br>0 No<br>1 Si<br><br>Custom alignment: RH |
| 22 | asma | Asma (diagnosticado por un médico) | radio<br>0 No<br>1 Si<br><br>Custom alignment: RH |
| 23 | enf_renal_cronica | Enfermedad renal crónica | radio<br>0 No<br>1 Si<br><br>Custom alignment: RH |
| 24 | fg_30<br>Show the field ONLY if:<br>[enf_renal_cronica] = '1' | FG < 30 | radio<br>0 No<br>1 Si<br><br>Custom alignment: RH |
| 25 | enf_hepatica_mod_grave | Enfermedad hepática moderada o grave | radio<br>0 No<br>1 Si<br><br>Custom alignment: RH |
| 26 | enf_hepatica_leve | Enfermedad hepatica leve | radio<br>0 No<br>1 Si<br><br>Custom alignment: RH |
| 27 | trast_neurologico_cronico | Trastorno neurológico crónico | radio<br>0 No<br>1 Si<br><br>Custom alignment: RH |

| | | | |
|---|---|---|---|
| 28 | neoplasia_maligna | Neoplasia maligna | radio<br>0 No<br>1 Si<br><br>Custom alignment: RH |
| 29 | enf_hematologica_cronica | Enfermedad hematológica crónica | radio<br>0 No<br>1 Si<br><br>Custom alignment: RH |
| 30 | sida_vih | SIDA/VIH | radio<br>0 No<br>1 Si<br><br>Custom alignment: RH |
| 31 | obesidad | Obesidad | radio<br>0 No<br>1 Si<br><br>Custom alignment: RH |
| 32 | diabetes | Diabetes | radio<br>0 No<br>1 Si<br><br>Custom alignment: RH |
| 33 | diabetes_con_compli | Diabetes con complicaciones | radio<br>0 No<br>1 Si<br><br>Custom alignment: RH |
| 34 | diabetes_sin_compli | Diabetes sin complicaciones | radio<br>0 No<br>1 Si<br><br>Custom alignment: RH |
| 35 | trast_reumatologico | Trastorno reumatológico | radio<br>0 No<br>1 Si<br><br>Custom alignment: RH |
| 36 | demencia | Demencia | radio<br>0 No<br>1 Si<br><br>Custom alignment: RH |
| 37 | malnutricion | Malnutrición | radio<br>0 No<br>1 Si<br><br>Custom alignment: RH |
| 38 | dislipemia | Dislipemia | radio<br>0 No<br>1 Si<br><br>Custom alignment: RH |
| 39 | trastorno_mental_comun | Trastorno mental comun | radio<br>0 No<br>1 Si<br><br>Custom alignment: RH |
| 40 | trastorno_mental_comun_espe<br><br>Show the field ONLY if:<br>[trastorno_mental_comun] = '1' | Trastorno mental comun | radio<br>1 Depresion<br>2 Ansiedad<br>3 Otro<br><br>Custom alignment: RH |

| | | | |
|---|---|---|---|
| 41 | trastorno_mental_grave | Trastorno mental grave | radio<br>0 No<br>1 Si<br><br>Custom alignment: RH |
| 42 | trastorno_mental_grave_2 | Trastorno mental grave | radio<br>1 Esquizofrenia<br>2 Trastorno bipolar<br>3 Otro<br><br>Custom alignment: RH |
| 43 | embarazo | Embarazo | radio<br>0 No<br>1 Si<br><br>Custom alignment: RH |
| 44 | post_parto_6m | Post-parto (< 6m) | radio<br>0 No<br>1 Si<br><br>Custom alignment: RH |
| 45 | fumador | Fumador | radio<br>0 No<br>1 Si<br><br>Custom alignment: RH |
| 46 | habito_tabaquico | Habito tabaquico | radio<br>0 Nunca<br>1 Exfumador<br>2 Fumador<br><br>Custom alignment: RH |
| 47 | indice_paquetes_anyo<br>Show the field ONLY if: [habito_tabaquico] = '1' or [habito_tabaquico] = '2' | Indice Paquetes Año | text (integer)<br>Custom alignment: RH |
| 48 | psoriasis | Psoriasis | radio<br>0 No<br>1 Si<br><br>Custom alignment: RH |
| 49 | dermatitis_atopica | Dermatitis atopica | radio<br>0 No<br>1 Si<br><br>Custom alignment: RH |
| 50 | hidradenitis | Hidradenitis | radio<br>0 No<br>1 Si<br><br>Custom alignment: RH |
| 51 | viajes_recientes | Viajes recientes | radio<br>0 No<br>1 Si<br><br>Custom alignment: RH |
| 52 | viajes_recientes_esp<br>Show the field ONLY if: [viajes_recientes] = '1' | Especificar | text<br>Custom alignment: RH |
| 53 | otros_factores_riesgo | Otros factores de riesgo relevantes | radio<br>0 No<br>1 Si<br><br>Custom alignment: RH |

| | | | |
|---|---|---|---|
| 54 | otros_factores_riesgo_espe<br>Show the field ONLY if: [otros_factores_riesgo] = '1' | Especificar | notes<br>Custom alignment: LV |
| 55 | charlson_comorbidity_index | Section Header:<br>Charlson Comorbidity Index (CCI) | text (integer, Min: 0, Max: 37)<br>Custom alignment: RH |
| 56 | antecedentes_personales_complete | Section Header: *Form Status*<br>Complete? | dropdown<br>0 Incomplete<br>1 Unverified<br>2 Complete |
| Instrument: **Variables Clinicas** (variables_clinicas) | | | |
| 57 | fecha_inicio_sintomas | Fecha de inicio de síntomas<br>*dd-mm-aaaa* | text (date_dmy) |
| 58 | fecha_llegada_urgencias | Fecha de llegada a urgencias<br>*dd-mm-aaaa hh:mm* | text (datetime_dmy) |
| 59 | fecha_alta_urgencias | Fecha de alta de urgencias<br>*dd-mm-aaaa hh:mm* | text (datetime_dmy) |
| 60 | fecha_hospitalizacion | Fecha de hospitalización<br>*dd-mm-aaaa hh:mm* | text (datetime_dmy) |
| 61 | necesidad_oxigenoterapia | Section Header:<br>Necesidad de oxigenoterapia | radio<br>0 No<br>1 Si<br>Custom alignment: RH |
| 62 | tipo_oxigenoterapia<br>Show the field ONLY if: [necesidad_oxigenoterapia] = '1' | Tipo | radio<br>1 Mascarilla Venturi<br>2 Mascarilla simple<br>3 Gafas nasales<br>4 Mascarilla con reservorio<br>5 Ventilación mecánica no invasiva<br>6 Ventilación mecánica invasiva<br>Custom alignment: LV |
| 63 | uso_pronos | Uso de pronos | radio<br>0 No<br>1 Si<br>Custom alignment: RH |
| 64 | peep | PEEP | text (number)<br>Custom alignment: RH |
| 65 | ipap | IPAP | text (number)<br>Custom alignment: RH |
| 66 | frecuencia_respiratoria | Frecuencia respiratoria<br>*rpm* | text (number)<br>Custom alignment: RH |
| 67 | tiempo_inspiratorio | Tiempo inspiratorio<br>*seg* | text (integer)<br>Custom alignment: RH |
| 68 | necesidad_ingreso_uci | Section Header:<br>Necesidad de ingreso en Unidad de Cuidados Intensivos (UCI) | radio<br>0 No<br>1 Si<br>Custom alignment: RH |
| 69 | disposicion_camas | Subsidiario pese a no disponer de camas | radio<br>0 No<br>1 Si<br>Custom alignment: RH |
| 70 | fecha_ingreso_uci<br>Show the field ONLY if: [necesidad_ingreso_uci] = '1' | Fecha de ingreso<br>*dd-mm-aaaa hh:mm* | text (datetime_dmy)<br>Custom alignment: RH |
| 71 | fecha_salida_uci<br>Show the field ONLY if: [necesidad_ingreso_uci] = '1' | Fecha de salida de UCI<br>*dd-mm-aaaa hh:mm* | text (datetime_dmy)<br>Custom alignment: RH |

| | | | |
|---|---|---|---|
| 72 | reingreso_uci | Reingreso en UCI | radio<br>0 No<br>1 Si<br><br>Custom alignment: RH |
| 73 | fecha_alta | Fecha de alta<br>*dd-mm-aaaa* | text (date_dmy)<br>Custom alignment: RH |
| 74 | exitus | Exitus | radio<br>0 No<br>1 Si<br><br>Custom alignment: RH |
| 75 | fecha_exitus<br><br>Show the field ONLY if:<br>[exitus] = '1' | Fecha de éxitus<br>*dd-mm-aaaa hh:mm* | text (date_dmy)<br>Custom alignment: RH |
| 76 | enfermedad_no_complicada | Section Header: *Nivel de gravedad en el momento del diagnóstico, según de la OMS del documento del 13 de marzo sobre manejo clínico*<br>Enfermedad no complicada | radio<br>0 No<br>1 Si<br><br>Custom alignment: RH |
| 77 | neumonia_leve | Neumonía leve | radio<br>0 No<br>1 Si<br><br>Custom alignment: RH |
| 78 | neumonia_grave | Neumonía grave | radio<br>0 No<br>1 Si<br><br>Custom alignment: RH |
| 79 | distres_respiratorio | Distrés respiratorio | radio<br>0 No<br>1 Si<br><br>Custom alignment: RH |
| 80 | sepsis | Sepsis | radio<br>0 No<br>1 Si<br><br>Custom alignment: RH |
| 81 | shock_septico | Shock séptico | radio<br>0 No<br>1 Si<br><br>Custom alignment: RH |
| 82 | variables_clinicas_complete | Section Header: *Form Status*<br>Complete? | dropdown<br>0 Incomplete<br>1 Unverified<br>2 Complete |
| Instrument: **Sintomas** (sintomas) | | | |
| 83 | fiebre | Fiebre | radio<br>0 No<br>1 Si<br><br>Custom alignment: RH |
| 84 | cefalea | Cefalea | radio<br>0 No<br>1 Si<br><br>Custom alignment: RH |
| 85 | malestar_general | Malestar general | radio<br>0 No<br>1 Si<br><br>Custom alignment: RH |

| | | | |
|---|---|---|---|
| 86 | mialgias | Mialgias o artralgias generalizadas | radio<br>0 No<br>1 Si<br><br>Custom alignment: RH |
| 87 | rinorrea | Rinorrea | radio<br>0 No<br>1 Si<br><br>Custom alignment: RH |
| 88 | disgeusia | Disgeusia | radio<br>0 No<br>1 Si<br><br>Custom alignment: RH |
| 89 | anosmia | Anosmia | radio<br>0 No<br>1 Si<br><br>Custom alignment: RH |
| 90 | tos | Tos | radio<br>0 No<br>1 Si<br><br>Custom alignment: RH |
| 91 | tos_productiva | Tos productiva | radio<br>0 No<br>1 Si<br><br>Custom alignment: RH |
| 92 | odinofagia | Odinofagia | radio<br>0 No<br>1 Si<br><br>Custom alignment: RH |
| 93 | dolor_toracico | Dolor toracico | radio<br>0 No<br>1 Si<br><br>Custom alignment: RH |
| 94 | dolor_costal | Dolor costal | radio<br>0 No<br>1 Si<br><br>Custom alignment: RH |
| 95 | expectoracion_hemoptoica | Expectoracion hemoptoica | radio<br>0 No<br>1 Si<br><br>Custom alignment: RH |
| 96 | disnea | Disnea | radio<br>0 No<br>1 Si<br><br>Custom alignment: RH |
| 97 | dolor_abdominal | Dolor abdominal | radio<br>0 No<br>1 Si<br><br>Custom alignment: RH |
| 98 | diarrea | Diarrea | radio<br>0 No<br>1 Si<br><br>Custom alignment: RH |

| | | | |
|---|---|---|---|
| 99 | nauseas | Nauseas | radio<br>0 No<br>1 Si<br><br>Custom alignment: RH |
| 100 | vomitos | Vomitos | radio<br>0 No<br>1 Si<br><br>Custom alignment: RH |
| 101 | alt_nivel_conciencia | Alteracion del nivel de conciencia | radio<br>0 No<br>1 Si<br><br>Custom alignment: RH |
| 102 | alt_nivel_comportamiento | Alteracion del comportamiento | radio<br>0 No<br>1 Si<br><br>Custom alignment: RH |
| 103 | convulsiones | Convulsiones | radio<br>0 No<br>1 Si<br><br>Custom alignment: RH |
| 104 | sintomas_complete | Section Header: *Form Status*<br>Complete? | dropdown<br>0 Incomplete<br>1 Unverified<br>2 Complete |
| Instrument: **Complicaciones** (complicaciones) | | | |
| 105 | infecciones_ingreso | Infecciones durante el ingreso | radio<br>0 No<br>1 Si<br><br>Custom alignment: RH |
| 106 | microorganismo_determinado | Microorganismo determinado | radio<br>0 No<br>1 Si<br><br>Custom alignment: RH |
| 107 | microorganismo<br>Show the field ONLY if:<br>[microorganismo_determinado] = '1' | Microorganismo | text<br>Custom alignment: RH |
| 108 | neumonia_bacteriana | Neumonia bacteriana sobreimpuesta | radio<br>0 No<br>1 Si<br><br>Custom alignment: RH |
| 109 | sdra | SDRA | radio<br>0 No<br>1 Si<br><br>Custom alignment: RH |
| 110 | neumotorax | Neumotorax | radio<br>0 No<br>1 Si<br><br>Custom alignment: RH |
| 111 | efusion_pleural | Efusion pleural | radio<br>0 No<br>1 Si<br><br>Custom alignment: RH |

| | | | |
|---|---|---|---|
| 112 | meningitis | Meningitis | radio<br>0 No<br>1 Si<br><br>Custom alignment: RH |
| 113 | convulsiones_compli | Convulsiones | radio<br>0 No<br>1 Si<br><br>Custom alignment: RH |
| 114 | accidente_cerebrovascular | Accidente cerebrovascular | radio<br>0 No<br>1 Si<br><br>Custom alignment: RH |
| 115 | insuf_cardiaca_congestiva | Insuficiencia cardiaca congestiva | radio<br>0 No<br>1 Si<br><br>Custom alignment: RH |
| 116 | miocarditis | Miocarditis | radio<br>0 No<br>1 Si<br><br>Custom alignment: RH |
| 117 | pericarditis | Pericarditis | radio<br>0 No<br>1 Si<br><br>Custom alignment: RH |
| 118 | endocarditis | Endocarditis | radio<br>0 No<br>1 Si<br><br>Custom alignment: RH |
| 119 | arritmia | Arritmia | radio<br>0 No<br>1 Si<br><br>Custom alignment: RH |
| 120 | isquemia_cardiaca | Isquemia cardiaca | radio<br>0 No<br>1 Si<br><br>Custom alignment: RH |
| 121 | parada_cardiaca | Parada cardiaca | radio<br>0 No<br>1 Si<br><br>Custom alignment: RH |
| 122 | bacteriemia | Bacteriemia | radio<br>0 No<br>1 Si<br><br>Custom alignment: RH |
| 123 | alteracion_coagulacion | Alteracion de la coagulacion | radio<br>0 No<br>1 Si<br><br>Custom alignment: RH |
| 124 | anemia_subsidiaria | Anemia subsidiaria de transfusion | radio<br>0 No<br>1 Si<br><br>Custom alignment: RH |

| | | | |
|---|---|---|---|
| 125 | rabdomiolisis | Rabdomiolisis | radio<br>0 No<br>1 Si<br><br>Custom alignment: RH |
| 126 | fallo_renal_agudo | Fallo renal agudo | radio<br>0 No<br>1 Si<br><br>Custom alignment: RH |
| 127 | hemorragia_digestiva | Hemorragia digestiva | radio<br>0 No<br>1 Si<br><br>Custom alignment: RH |
| 128 | pancreatitis | Pancreatitis | radio<br>0 No<br>1 Si<br><br>Custom alignment: RH |
| 129 | fallo_hepatico | Fallo hepatico | radio<br>0 No<br>1 Si<br><br>Custom alignment: RH |
| 130 | sind_confusional_agudo | Sinrdrome confusional agudo/delirium | radio<br>0 No<br>1 Si<br><br>Custom alignment: RH |
| 131 | compl_psiquiatricas | Complicaciones psiquiatricas | radio<br>0 No<br>1 Si<br><br>Custom alignment: RH |
| 132 | reaccion_adversa_med | Reaccion Adversa a Medicamentos | radio<br>0 No<br>1 Si<br><br>Custom alignment: RH |
| 133 | reaccion_adversa_med_grave<br>Show the field ONLY if:<br>[reaccion_adversa_med] = '1' | Reaccion Adversa a Medicamentos Grave | radio<br>0 No<br>1 Si<br><br>Custom alignment: RH |
| 134 | medicamento_reaccion<br>Show the field ONLY if:<br>[reaccion_adversa_med] = '1' | Medicamento(s) | text<br>Custom alignment: RH |
| 135 | otras_complicaciones | Otras complicaciones | text<br>Custom alignment: RH |
| 136 | complicaciones_complete | Section Header: *Form Status*<br>Complete? | dropdown<br>0 Incomplete<br>1 Unverified<br>2 Complete |
| Instrument: **Evolucion** (evolucion) | | | |
| 137 | negativo_confirmado_pcr | Negativo confirmado por PCR | radio<br>0 No<br>1 Si<br><br>Custom alignment: RH |
| 138 | fecha_negativo<br>Show the field ONLY if:<br>[negativo_confirmado_pcr] = '1' | Fecha negativo<br>*dd-mm-aaaa* | text (date_mdy)<br>Custom alignment: RH |

| | | | |
|---|---|---|---|
| 139 | evolucion_complete | Section Header: *Form Status*<br>Complete? | dropdown<br>0 Incomplete<br>1 Unverified<br>2 Complete |
| Instrument: **Escalas** (escalas) | | | |
| 140 | escala_curb_65 | Escala CURB-65 | text (integer, Min: 0, Max: 5)<br>Custom alignment: RH |
| 141 | escala_fine | ESCALA FINE | text (integer, Min: 0, Max: 5)<br>Custom alignment: RH |
| 142 | escala_qsofa | Escala qSOFA | text (integer, Min: 0, Max: 3) |
| 143 | escala_sofa | Escala SOFA | text (integer, Min: 0, Max: 24) |
| 144 | psi | PSI | text (integer) |
| 145 | escalas_complete | Section Header: *Form Status*<br>Complete? | dropdown<br>0 Incomplete<br>1 Unverified<br>2 Complete |
| Instrument: **Medicacion Habitual** (medicacion_habitual) | | | |
| 146 | farmaco_mh | Farmaco | text |
| 147 | indicacion_mh | Indicacion | text |
| 148 | forma_farmaceutica_mh | Forma Farmaceutica | text |
| 149 | dosis_total_diaria_mh | Dosis Total Diaria | text |
| 150 | via_admin_mh | Via de Administracion | dropdown<br>IM Intramuscular<br>IV Intravenosa<br>VB Via bucal (enjuages, aplicacion topica)<br>VIC Via intracavernosa<br>VINH Via inhalatoria<br>VO Via oral<br>VOF Via oftálmica<br>VOT Via otica<br>VP Via parenteral<br>VR Via rectal<br>VSC Via subcutánea<br>VSI Via sublingual<br>VT Via tópica<br>VTD Via trandermica<br>VV Via vaginal o vulvar |
| 151 | fecha_inicio_mh | Fecha de inicio | radio<br>0 No disponible<br>1 Fecha de inicio conocida o aproximada |
| 152 | fecha_inicio_conocida_mh<br>Show the field ONLY if:<br>[fecha_inicio_mh] = '1' | Fecha de inicio<br>*dd-mm-aaaa* | text (date_dmy)<br>Custom alignment: RH |
| 153 | fecha_fin_mh | Fecha de fin | radio<br>0 Continua<br>1 Fecha de fin conocida o aproximada |
| 154 | fecha_fin_conocida_mh<br>Show the field ONLY if:<br>[fecha_fin_mh] = '1' | Fecha de fin<br>*dd-mm-aaaa* | text (date_dmy)<br>Custom alignment: RH |
| 155 | medicacion_habitual_complete | Section Header: *Form Status*<br>Complete? | dropdown<br>0 Incomplete<br>1 Unverified<br>2 Complete |
| Instrument: **Medicacion durante el Episodio** (medicacion_durante_el_episodio) | | | |
| 156 | farmaco_mc | Farmaco | text |
| 157 | indicacion_mc | Indicacion | text |

| | | | |
|---|---|---|---|
| 158 | forma_farmaceutica_mc | Forma Farmaceutica | text |
| 159 | dosis_diaria_total_mc | Dosis Diaria Total | text |
| 160 | via_admin_mc | Via de Administracion | dropdown<br>IM Intramuscular<br>IV Intravenosa<br>VB Via bucal (enjuages, aplicacion topica)<br>VIC Via intracavernosa<br>VINH Via inhalatoria<br>VO Via oral<br>VOF Via oftálmica<br>VOT Via otica<br>VP Via parenteral<br>VR Via rectal<br>VSC Via subcutánea<br>VSI Via sublingual<br>VT Via tópica<br>VTD Via trandermica<br>VV Via vaginal o vulvar |
| 161 | fecha_inicio_mc | Fecha de inicio | radio<br>0 No disponible<br>1 Fecha de inicio conocida o aproximada |
| 162 | fecha_inicio_conocida_mc<br>Show the field ONLY if:<br>[fecha_inicio_mc] = '1' | Fecha de inicio<br>*dd-mm-aaaa* | text (date_dmy)<br>Custom alignment: RH |
| 163 | fecha_fin_mc | Fecha de fin | radio<br>0 Continua<br>1 Fecha de fin conocida o aproximada |
| 164 | fecha_fin_conocida_mc<br>Show the field ONLY if:<br>[fecha_fin_mc] = '1' | Fecha de fin<br>*dd-mm-aaaa* | text (date_dmy)<br>Custom alignment: RH |
| 165 | medicacion_durante_el_episodio_complete | Section Header: *Form Status*<br>Complete? | dropdown<br>0 Incomplete<br>1 Unverified<br>2 Complete |
| Instrument: **Constantes** (constantes) | | | |
| 166 | fecha_constantes | Fecha<br>*dd-mm-aaaa hh:mm* | text (datetime_dmy) |
| 167 | temperatura | Temperatura<br>*ºC* | text (number) |
| 168 | frec_cardiaca | Frecuencia Cardiaca<br>*lpm* | text (number) |
| 169 | frec_respiratoria | Frecuencia Respiratoria<br>*rpm* | text (number) |
| 170 | tension_arterial_sistolica | Tension arterial - Sistolica<br>*mmHg* | text (integer) |
| 171 | tension_arterial_diastolica | Tension arterial - Diastolica<br>*mmHg* | text (integer) |
| 172 | sato2_basal | SatO2 basal<br>*%* | text (integer) |
| 173 | sato2_o2 | SatO2 con O2<br>*%* | text (integer) |
| 174 | constantes_complete | Section Header: *Form Status*<br>Complete? | dropdown<br>0 Incomplete<br>1 Unverified<br>2 Complete |
| Instrument: **Serologias** (serologias) | | | |

| | | | |
|---|---|---|---|
| 175 | vih | VIH | radio<br>0 No<br>1 Si<br><br>Custom alignment: RH |
| 176 | fecha_vih<br><br>Show the field ONLY if:<br>[vih] = '1' | Fecha<br>*dd-mm-aaaa* | text (date_dmy) |
| 177 | vhc | VHC | radio<br>0 No<br>1 Si<br><br>Custom alignment: RH |
| 178 | fecha_vhc<br><br>Show the field ONLY if:<br>[vhc] = '1' | Fecha<br>*dd-mm-aaaa* | text (date_dmy) |
| 179 | vhb | VHB | radio<br>0 No<br>1 Si<br><br>Custom alignment: RH |
| 180 | fecha_vhb<br><br>Show the field ONLY if:<br>[vhb] = '1' | Fecha<br>*dd-mm-aaaa* | text (date_dmy) |
| 181 | serologias_complete | Section Header: *Form Status*<br>Complete? | dropdown<br>0 Incomplete<br>1 Unverified<br>2 Complete |
| Instrument: **PCR** (pcr) | | | |
| 182 | fecha_pcr_sars_cov2 | Fecha de PCR SARS-CoV-2<br>*dd-mm-aaaa hh:mm* | text (datetime_dmy) |
| 183 | pcr_sars_covid2_num | PCR SARS-CoV-2 - Carga viral<br>*copias/mL* | text (number)<br>Custom alignment: RH |
| 184 | resultado_pcr_sars_cov2 | Resultado PCR SARS-CoV-2 | radio<br>0 Negativo<br>1 Positivo<br><br>Custom alignment: RH |
| 185 | pcr_sars_cov2_numero_test | Número de test realizados | text (integer) |
| 186 | pcr_complete | Section Header: *Form Status*<br>Complete? | dropdown<br>0 Incomplete<br>1 Unverified<br>2 Complete |
| Instrument: **Analitica y Laboratorio** (analitica_y_laboratorio) | | | |
| 187 | fecha_analitica | Fecha Analitica<br>*dd-mm-aaaa hh:mm* | text (datetime_dmy) |
| 188 | ddimero | Section Header:<br>D-Dímero<br>*ng/mL* | text (number)<br>Custom alignment: RH |
| 189 | pcr | PCR<br>*mg/L* | text (number)<br>Custom alignment: RH |
| 190 | vsg | VSG<br>*mm/h* | text (number)<br>Custom alignment: RH |
| 191 | leucocitos_nr | Section Header: *HEMOGRAMA*<br>Leucocitos | radio<br>0 No Reallizado<br>1 Realizado<br><br>Custom alignment: RH |
| 192 | leucocitos<br><br>Show the field ONLY if:<br>[leucocitos_nr] = '1' | Leucocitos | text (number)<br>Custom alignment: RH |

| | | | |
|---|---|---|---|
| 193 | leucocitos_uds<br>Show the field ONLY if:<br>[leucocitos_nr] = 1 | Unidades | radio<br>1 x103/uL<br>2 x109/L<br>9 Otras<br>Custom alignment: RH |
| 194 | leucocitos_uds_otras<br>Show the field ONLY if:<br>[leucocitos_uds] = 9 | Otras unidades | text<br>Custom alignment: RH |
| 195 | linfocitos_nr | Linfocitos | radio<br>0 No Reallizado<br>1 Realizado<br>Custom alignment: RH |
| 196 | linfocitos<br>Show the field ONLY if:<br>[linfocitos_nr] = 1 | Linfocitos | text (number)<br>Custom alignment: RH |
| 197 | linfocitos_uds<br>Show the field ONLY if:<br>[linfocitos_nr] = 1 | Unidades | radio<br>1 %<br>2 x103/uL<br>3 x109/L<br>9 Otras<br>Custom alignment: RH |
| 198 | linfocitos_uds_otras<br>Show the field ONLY if:<br>[linfocitos_uds] = 9 | Otras unidades | text<br>Custom alignment: RH |
| 199 | neutrofilos_nr | Neutrofilos | radio<br>0 No Reallizado<br>1 Realizado<br>Custom alignment: RH |
| 200 | neutrofilos<br>Show the field ONLY if:<br>[neutrofilos_nr] = 1 | Neutrofios | text (number)<br>Custom alignment: RH |
| 201 | neutrofilos_uds<br>Show the field ONLY if:<br>[neutrofilos_nr] = 1 | Unidades | radio<br>1 %<br>2 x103/uL<br>3 x109/L<br>9 Otras<br>Custom alignment: RH |
| 202 | neutrofilos_uds_otras<br>Show the field ONLY if:<br>[neutrofilos_uds] = 9 | Otras unidades | text<br>Custom alignment: RH |
| 203 | vcm_nr | Volumen Corpuscular Medio | radio<br>0 No Reallizado<br>1 Realizado<br>Custom alignment: RH |
| 204 | vcm<br>Show the field ONLY if:<br>[vcm_nr] = 1 | Volumen Corpuscular Medio | text (number)<br>Custom alignment: RH |
| 205 | vcm_uds<br>Show the field ONLY if:<br>[vcm_nr] = 1 | Unidades | radio<br>1 mm3<br>2 fL<br>9 Otras<br>Custom alignment: RH |
| 206 | vcm_uds_otras<br>Show the field ONLY if:<br>[vcm_uds] = 9 | Otras unidades | text<br>Custom alignment: RH |

| | | | |
|---|---|---|---|
| 207 | hemoglobina_nr | Hemoglobina | radio<br>0 No Reallizado<br>1 Realizado<br><br>Custom alignment: RH |
| 208 | hemoglobina<br><br>Show the field ONLY if:<br>[hemoglobina_nr] = 1 | Hemoglobina | text (number)<br>Custom alignment: RH |
| 209 | hemoglobina_uds<br><br>Show the field ONLY if:<br>[hemoglobina_nr] = 1 | Unidades | radio<br>1 g/dL<br>2 g/L<br>9 Otras<br><br>Custom alignment: RH |
| 210 | hemoglobina_uds_otras<br><br>Show the field ONLY if:<br>[hemoglobina_uds] = 9 | Otras unidades | text<br>Custom alignment: RH |
| 211 | hematocrito_nr | Hematocrito | radio<br>0 No Reallizado<br>1 Realizado<br><br>Custom alignment: RH |
| 212 | hematocrito<br><br>Show the field ONLY if:<br>[hematocrito_nr] = 1 | Hematocrito | text (number)<br>Custom alignment: RH |
| 213 | hematocrito_uds<br><br>Show the field ONLY if:<br>[hematocrito_nr] = 1 | Unidades | radio<br>1 %<br>9 Otras<br><br>Custom alignment: RH |
| 214 | hematocrito_uds_otras<br><br>Show the field ONLY if:<br>[hematocrito_uds] = 9 | Otras unidades | text<br>Custom alignment: RH |
| 215 | hematies_nr | Hematies | radio<br>0 No Reallizado<br>1 Realizado<br><br>Custom alignment: RH |
| 216 | hematies<br><br>Show the field ONLY if:<br>[hematies_nr] = 1 | Hematies | text (number)<br>Custom alignment: RH |
| 217 | hematies_uds<br><br>Show the field ONLY if:<br>[hematies_nr] = 1 | Unidades | radio<br>1 x106<br>9 Otras<br><br>Custom alignment: RH |
| 218 | hematies_uds_otras<br><br>Show the field ONLY if:<br>[hematies_uds] = 9 | Otras unidades | text<br>Custom alignment: RH |
| 219 | plaquetas_nr | Plaquetas | radio<br>0 No Reallizado<br>1 Realizado<br><br>Custom alignment: RH |
| 220 | plaquetas<br><br>Show the field ONLY if:<br>[plaquetas_nr] = 1 | Plaquetas | text (number)<br>Custom alignment: RH |
| 221 | plaquetas_uds<br><br>Show the field ONLY if:<br>[plaquetas_nr] = 1 | Unidades | radio<br>1 x103/uL<br>2 x109/L<br>9 Otras<br><br>Custom alignment: RH |

| | | | |
|---|---|---|---|
| 222 | plaquetas_uds_otras<br>Show the field ONLY if:<br>[plaquetas_uds] = 9 | Otras unidades | text<br>Custom alignment: RH |
| 223 | act_protombina_nr | Section Header: *COAGULACION*<br>Actividad de Protombina | radio<br>0 No Reallizado<br>1 Realizado<br>Custom alignment: RH |
| 224 | act_protombina<br>Show the field ONLY if:<br>[act_protombina_nr] = 1 | Actividad de Protombina | text (number)<br>Custom alignment: RH |
| 225 | act_protombina_uds<br>Show the field ONLY if:<br>[act_protombina_nr] = 1 | Unidades | radio<br>1 %<br>9 Otras<br>Custom alignment: RH |
| 226 | act_protombina_uds_otras<br>Show the field ONLY if:<br>[act_protombina_uds] = 9 | Otras Unidades | text<br>Custom alignment: RH |
| 227 | tp_nr | Tiempo de Protombina (TP) | radio<br>0 No Reallizado<br>1 Realizado<br>Custom alignment: RH |
| 228 | tp<br>Show the field ONLY if:<br>[tp_nr] = 1 | Tiempo de Protombina (TP) | text (number)<br>Custom alignment: RH |
| 229 | tp_uds<br>Show the field ONLY if:<br>[tp_nr] = 1 | Unidades | radio<br>1 seg<br>9 Otras<br>Custom alignment: RH |
| 230 | tp_uds_otras<br>Show the field ONLY if:<br>[tp_uds] = 9 | Otras Unidades | text<br>Custom alignment: RH |
| 231 | inr_nr | INR | radio<br>0 No Reallizado<br>1 Realizado<br>Custom alignment: RH |
| 232 | inr<br>Show the field ONLY if:<br>[inr_nr] = 1 | INR | text (number)<br>Custom alignment: RH |
| 233 | ttpa_nr | Tiempo de Tromboplastina parcial activado (TTPa) | radio<br>0 No Reallizado<br>1 Realizado<br>Custom alignment: RH |
| 234 | ttpa<br>Show the field ONLY if:<br>[ttpa_nr] = 1 | Tiempo de Tromboplastina parcial activado (TTPa) | text (number)<br>Custom alignment: RH |
| 235 | ttpa_uds<br>Show the field ONLY if:<br>[ttpa_nr] = 1 | Unidades | radio<br>1 seg<br>9 Otras<br>Custom alignment: RH |
| 236 | ttpa_uds_otras<br>Show the field ONLY if:<br>[ttpa_uds] = 9 | Otras Unidades | text<br>Custom alignment: RH |
| 237 | tt_nr | Tiempo de Trombina (TT) | radio<br>0 No Reallizado<br>1 Realizado<br>Custom alignment: RH |

| | | | |
|---|---|---|---|
| 238 | tt<br>Show the field ONLY if:<br>[tt_nr] = 1 | Tiempo de Trombina (TT) | text (number)<br>Custom alignment: RH |
| 239 | tt_uds<br>Show the field ONLY if:<br>[tt_nr] = 1 | Unidades | radio<br>1 seg<br>9 Otras<br>Custom alignment: RH |
| 240 | tt_uds_otras<br>Show the field ONLY if:<br>[tt_uds] = 9 | Otras Unidades | text<br>Custom alignment: RH |
| 241 | fibrinogeno_nr | Fibrinogeno | radio<br>0 No Reallizado<br>1 Realizado<br>Custom alignment: RH |
| 242 | fibrinogeno<br>Show the field ONLY if:<br>[fibrinogeno_nr] = 1 | Fibrinogeno | text (number)<br>Custom alignment: RH |
| 243 | fibrinogeno_uds<br>Show the field ONLY if:<br>[fibrinogeno_nr] = 1 | Unidades | radio<br>1 g/L<br>2 mg/dL<br>9 Otras<br>Custom alignment: RH |
| 244 | fibrinogeno_uds_otras<br>Show the field ONLY if:<br>[fibrinogeno_uds] = 9 | Otras Unidades | text<br>Custom alignment: RH |
| 245 | glucosa_nr | Section Header: *BIOQUIMICA*<br>Glucosa | radio<br>0 No Reallizado<br>1 Realizado<br>Custom alignment: RH |
| 246 | glucosa<br>Show the field ONLY if:<br>[glucosa_nr] = 1 | Glucosa | text (number)<br>Custom alignment: RH |
| 247 | glucosa_uds<br>Show the field ONLY if:<br>[glucosa_nr] = 1 | Unidades | radio<br>1 mmol/L<br>2 mg/dL<br>9 Otras<br>Custom alignment: RH |
| 248 | glucosa_uds_otras<br>Show the field ONLY if:<br>[glucosa_uds] = 9 | Otras Unidades | text<br>Custom alignment: RH |
| 249 | colesterol_nr | Colesterol | radio<br>0 No Reallizado<br>1 Realizado<br>Custom alignment: RH |
| 250 | colesterol<br>Show the field ONLY if:<br>[colesterol_nr]= 1 | Colesterol | text (number)<br>Custom alignment: RH |
| 251 | colesterol_uds<br>Show the field ONLY if:<br>[colesterol_nr] = 1 | Unidades | radio<br>1 mg/dL<br>9 Otras<br>Custom alignment: RH |
| 252 | colesterol_uds_otras<br>Show the field ONLY if:<br>[colesterol_uds] = 9 | Otras Unidades | text<br>Custom alignment: RH |
| 253 | hdl_nr | HDL | radio<br>0 No Reallizado<br>1 Realizado<br>Custom alignment: RH |

| | | | |
|---|---|---|---|
| 254 | hdl<br>Show the field ONLY if:<br>[hdl_nr] = 1 | HDL | text (number)<br>Custom alignment: RH |
| 255 | hdl_uds<br>Show the field ONLY if:<br>[hdl_nr] = 1 | Unidades | radio<br>1 mg/dL<br>9 Otras<br>Custom alignment: RH |
| 256 | hdl_uds_otras<br>Show the field ONLY if:<br>[hdl_uds] = 9 | Otras Unidades | text<br>Custom alignment: RH |
| 257 | ldl_nr | LDL | radio<br>0 No Reallizado<br>1 Realizado<br>Custom alignment: RH |
| 258 | ldl<br>Show the field ONLY if:<br>[ldl_nr] = 1 | LDL | text (number)<br>Custom alignment: RH |
| 259 | ldl_uds<br>Show the field ONLY if:<br>[ldl_nr] = 1 | Unidades | radio<br>1 mg/dL<br>9 Otras<br>Custom alignment: RH |
| 260 | ldl_uds_otras<br>Show the field ONLY if:<br>[ldl_uds] = 9 | Otras Unidades | text<br>Custom alignment: RH |
| 261 | trigliceridos_nr | Trigliceridos | radio<br>0 No Reallizado<br>1 Realizado<br>Custom alignment: RH |
| 262 | trigliceridos<br>Show the field ONLY if:<br>[trigliceridos_nr] = 1 | Trigliceridos | text (number)<br>Custom alignment: RH |
| 263 | trigliceridos_uds<br>Show the field ONLY if:<br>[trigliceridos_nr] = 1 | Unidades | radio<br>1 mmol/L<br>2 mg/dL<br>9 Otras<br>Custom alignment: RH |
| 264 | trigliceridos_uds_otras<br>Show the field ONLY if:<br>[trigliceridos_uds] = 9 | Otras Unidades | text<br>Custom alignment: RH |
| 265 | astgot_nr | AST/GOT | radio<br>0 No Reallizado<br>1 Realizado<br>Custom alignment: RH |
| 266 | astgot<br>Show the field ONLY if:<br>[astgot_nr] = 1 | AST/GOT | text (number)<br>Custom alignment: RH |
| 267 | astgot_uds<br>Show the field ONLY if:<br>[astgot_nr] = 1 | Unidades | radio<br>1 UI/L<br>9 Otras<br>Custom alignment: RH |
| 268 | astgot_uds_otras<br>Show the field ONLY if:<br>[astgot_uds] = 9 | Otras Unidades | text<br>Custom alignment: RH |
| 269 | altgpt_nr | ALT/GPT | radio<br>0 No Reallizado<br>1 Realizado<br>Custom alignment: RH |

| | | | |
|---|---|---|---|
| 270 | altgpt<br><br>Show the field ONLY if:<br>[altgpt_nr] = 1 | ALT/GPT | text (number)<br>Custom alignment: RH |
| 271 | altgpt_uds<br><br>Show the field ONLY if:<br>[altgpt_nr] = 1 | Unidades | radio<br>1 UI/L<br>9 Otras<br><br>Custom alignment: RH |
| 272 | altgpt_uds_otras<br><br>Show the field ONLY if:<br>[altgpt_uds] = 9 | Otras Unidades | text<br>Custom alignment: RH |
| 273 | ggt_nr | GGT | radio<br>0 No Reallizado<br>1 Realizado<br><br>Custom alignment: RH |
| 274 | ggt<br><br>Show the field ONLY if:<br>[ggt_nr] = 1 | GGT | text (number)<br>Custom alignment: RH |
| 275 | ggt_uds<br><br>Show the field ONLY if:<br>[ggt_nr] = 1 | Unidades | radio<br>1 UI/L<br>9 Otras<br><br>Custom alignment: RH |
| 276 | ggt_uds_otras<br><br>Show the field ONLY if:<br>[ggt_uds] = 9 | Otras Unidades | text<br>Custom alignment: RH |
| 277 | fosfatasa_alcalina_nr | Fosfatasa Alcalina | radio<br>0 No Reallizado<br>1 Realizado<br><br>Custom alignment: RH |
| 278 | fosfatasa_alcalina<br><br>Show the field ONLY if:<br>[fosfatasa_alcalina_nr] = 1 | Fosfatasa Alcalina | text (number)<br>Custom alignment: RH |
| 279 | fosfatasa_alcalina_uds<br><br>Show the field ONLY if:<br>[fosfatasa_alcalina_nr] = 1 | Unidades | radio<br>1 UI/L<br>9 Otras<br><br>Custom alignment: RH |
| 280 | fosfatasa_alcalina_uds_otras<br><br>Show the field ONLY if:<br>[fosfatasa_alcalina_uds] = 9 | Otras Unidades | text<br>Custom alignment: RH |
| 281 | bilirrubina_total_nr | Bilirrubina Total | radio<br>0 No Reallizado<br>1 Realizado<br><br>Custom alignment: RH |
| 282 | bilirrubina_total<br><br>Show the field ONLY if:<br>[bilirrubina_total_nr] = 1 | Bilirrubina Total | text (number)<br>Custom alignment: RH |
| 283 | bilirrubina_total_uds<br><br>Show the field ONLY if:<br>[bilirrubina_total_nr] = 1 | Unidades | radio<br>1 mg/dL<br>2 umol/L<br>9 Otras<br><br>Custom alignment: RH |
| 284 | bilirrubina_total_uds_otras<br><br>Show the field ONLY if:<br>[bilirrubina_total_uds] = 9 | Otras Unidades | text<br>Custom alignment: RH |

| | | | |
|---|---|---|---|
| 285 | creatinina_nr | Creatinina | radio<br>0 No Reallizado<br>1 Realizado<br><br>Custom alignment: RH |
| 286 | creatinina<br><br>Show the field ONLY if:<br>[creatinina_nr] = 1 | Creatinina | text (number)<br>Custom alignment: RH |
| 287 | creatinina_uds<br><br>Show the field ONLY if:<br>[creatinina_nr] = 1 | Unidades | radio<br>1 mg/dL<br>2 umol/L<br>9 Otras<br><br>Custom alignment: RH |
| 288 | creatinina_uds_otras<br><br>Show the field ONLY if:<br>[creatinina_uds] = 9 | Otras Unidades | text<br>Custom alignment: RH |
| 289 | sodio_nr | Sodio | radio<br>0 No Reallizado<br>1 Realizado<br><br>Custom alignment: RH |
| 290 | sodio<br><br>Show the field ONLY if:<br>[sodio_nr] = 1 | Sodio | text (number)<br>Custom alignment: RH |
| 291 | sodio_uds<br><br>Show the field ONLY if:<br>[sodio_nr] = 1 | Unidades | radio<br>1 mmol/L<br>2 mEq/L<br>9 Otras<br><br>Custom alignment: RH |
| 292 | sodio_uds_otras<br><br>Show the field ONLY if:<br>[sodio_uds] = 9 | Otras Unidades | text<br>Custom alignment: RH |
| 293 | potasio_nr | Potasio | radio<br>0 No Reallizado<br>1 Realizado<br><br>Custom alignment: RH |
| 294 | potasio<br><br>Show the field ONLY if:<br>[potasio_nr] = 1 | Potasio | text (number)<br>Custom alignment: RH |
| 295 | potasio_uds<br><br>Show the field ONLY if:<br>[potasio_nr] = 1 | Unidades | radio<br>1 mmol/L<br>2 mEq/L<br>9 Otras<br><br>Custom alignment: RH |
| 296 | potasio_uds_otras<br><br>Show the field ONLY if:<br>[potasio_uds] = 9 | Otras Unidades | text<br>Custom alignment: RH |
| 297 | proteina_nr | Proteina | radio<br>0 No Reallizado<br>1 Realizado<br><br>Custom alignment: RH |
| 298 | proteina<br><br>Show the field ONLY if:<br>[proteina_nr] = 1 | Proteina | text (number)<br>Custom alignment: RH |
| 299 | proteina_uds<br><br>Show the field ONLY if:<br>[proteina_nr] = 1 | Unidades | radio<br>1 g/dL<br>2 g/L<br>9 Otras<br><br>Custom alignment: RH |

| | | | |
|---|---|---|---|
| 300 | proteina_uds_otras<br>Show the field ONLY if:<br>[proteina_uds] = 9 | Otras Unidades | text<br>Custom alignment: RH |
| 301 | calcio_nr | Calcio | radio<br>0 No Reallizado<br>1 Realizado<br>Custom alignment: RH |
| 302 | calcio<br>Show the field ONLY if:<br>[calcio_nr] = 1 | Calcio | text (number)<br>Custom alignment: RH |
| 303 | calcio_uds<br>Show the field ONLY if:<br>[calcio_nr] = 1 | Unidades | radio<br>1 mmol/L<br>2 mg/dL<br>9 Otras<br>Custom alignment: RH |
| 304 | calcio_uds_otras<br>Show the field ONLY if:<br>[calcio_uds] = 9 | Otras Unidades | text<br>Custom alignment: RH |
| 305 | urea_nr | Urea | radio<br>0 No Reallizado<br>1 Realizado<br>Custom alignment: RH |
| 306 | urea<br>Show the field ONLY if:<br>[urea_nr] = 1 | Urea | text (number)<br>Custom alignment: RH |
| 307 | urea_uds<br>Show the field ONLY if:<br>[urea_nr] = 1 | Unidades | radio<br>1 mg/dL<br>9 Otras<br>Custom alignment: RH |
| 308 | urea_uds_otras<br>Show the field ONLY if:<br>[urea_uds] = 9 | Otras Unidades | text<br>Custom alignment: RH |
| 309 | hierro_nr | Hierro | radio<br>0 No Reallizado<br>1 Realizado<br>Custom alignment: RH |
| 310 | hierro<br>Show the field ONLY if:<br>[hierro_nr] = 1 | Hierro | text (number)<br>Custom alignment: RH |
| 311 | hierro_uds<br>Show the field ONLY if:<br>[hierro_nr] = 1 | Unidades | radio<br>1 ug/dL<br>2 umol/L<br>9 Otras<br>Custom alignment: RH |
| 312 | hierro_uds_otras<br>Show the field ONLY if:<br>[hierro_uds] = 9 | Otras Unidades | text<br>Custom alignment: RH |
| 313 | transferina_nr | Transferina | radio<br>0 No Reallizado<br>1 Realizado<br>Custom alignment: RH |
| 314 | transferina<br>Show the field ONLY if:<br>[transferina_nr] = 1 | Transferina | text (number)<br>Custom alignment: RH |

| | | | |
|---|---|---|---|
| 315 | transferina_uds<br>Show the field ONLY if:<br>[transferina_nr] = 1 | Unidades | radio<br>1 mg/dL<br>2 g/L<br>9 Otras<br>Custom alignment: RH |
| 316 | transferina_uds_otras<br>Show the field ONLY if:<br>[transferina_uds] = 9 | Otras Unidades | text<br>Custom alignment: RH |
| 317 | is_transferina_nr | IS Transferina | radio<br>0 No Reallizado<br>1 Realizado<br>Custom alignment: RH |
| 318 | is_transferina<br>Show the field ONLY if:<br>[is_transferina_nr] = 1 | IS Transferina | text (number)<br>Custom alignment: RH |
| 319 | is_transferina_uds<br>Show the field ONLY if:<br>[is_transferina_nr] = 1 | Unidades | radio<br>1 %<br>9 Otras<br>Custom alignment: RH |
| 320 | is_transferina_uds_otras<br>Show the field ONLY if:<br>[is_transferina_uds] = 9 | Otras Unidades | text<br>Custom alignment: RH |
| 321 | ferritina_nr | Ferritina | radio<br>0 No Reallizado<br>1 Realizado<br>Custom alignment: RH |
| 322 | ferritina<br>Show the field ONLY if:<br>[ferritina_nr] = 1 | Ferritina | text (number)<br>Custom alignment: RH |
| 323 | ferritina_uds<br>Show the field ONLY if:<br>[ferritina_nr] = 1 | Unidades | radio<br>1 ug/L<br>2 ng/mL<br>9 Otras<br>Custom alignment: RH |
| 324 | ferritina_uds_otras<br>Show the field ONLY if:<br>[ferritina_uds] = 9 | Otras Unidades | text<br>Custom alignment: RH |
| 325 | albumina_nr | Albumina | radio<br>0 No Reallizado<br>1 Realizado<br>Custom alignment: RH |
| 326 | albumina<br>Show the field ONLY if:<br>[albumina_nr] = 1 | Albumina | text (number) |
| 327 | albumina_uds<br>Show the field ONLY if:<br>[albumina_nr] = 1 | Unidades | radio<br>1 g/dL<br>2 g/L<br>9 Otras<br>Custom alignment: RH |
| 328 | albumina_uds_otras<br>Show the field ONLY if:<br>[albumina_uds] = 9 | Otras Unidades | text<br>Custom alignment: RH |
| 329 | troponina_t_nr | Section Header:<br>Troponina T | radio<br>0 No Reallizado<br>1 Realizado<br>Custom alignment: RH |

| | | | |
|---|---|---|---|
| 330 | troponina_t<br>Show the field ONLY if:<br>[troponina_t_nr] = 1 | Troponina T | text (number)<br>Custom alignment: RH |
| 331 | troponina_t_uds<br>Show the field ONLY if:<br>[troponina_t_nr] = 1 | Unidades | radio<br>1 ng/mL<br>2 ug/L<br>9 Otras<br>Custom alignment: RH |
| 332 | troponina_t_uds_otras<br>Show the field ONLY if:<br>[troponina_t_uds] = 9 | Otras Unidades | text<br>Custom alignment: RH |
| 333 | troponina_i_nr | Troponina I | radio<br>0 No Reallizado<br>1 Realizado<br>Custom alignment: RH |
| 334 | troponina_i<br>Show the field ONLY if:<br>[troponina_i_nr] = 1 | Troponina I | text (number)<br>Custom alignment: RH |
| 335 | troponina_i_uds<br>Show the field ONLY if:<br>[troponina_i_nr] = 1 | Unidades | radio<br>1 ng/mL<br>2 ug/L<br>9 Otras<br>Custom alignment: RH |
| 336 | troponina_i_uds_otras<br>Show the field ONLY if:<br>[troponina_i_uds] = 9 | Otras Unidades | text<br>Custom alignment: RH |
| 337 | ph_nr | Section Header: *GASOMETRIA ARTERIAL*<br>pH | radio<br>0 No Reallizado<br>1 Realizado<br>Custom alignment: RH |
| 338 | ph<br>Show the field ONLY if:<br>[ph_nr] = 1 | pH | text (number, Min: 6.75, Max: 8.25)<br>Custom alignment: RH |
| 339 | po2_nr | pO2 | radio<br>0 No Reallizado<br>1 Realizado<br>Custom alignment: RH |
| 340 | po2<br>Show the field ONLY if:<br>[po2_nr] = 1 | pO2 | text (number)<br>Custom alignment: RH |
| 341 | po2_uds<br>Show the field ONLY if:<br>[po2_nr] = 1 | Unidades | radio<br>1 mmHg<br>2 kPa<br>9 Otras<br>Custom alignment: RH |
| 342 | po2_uds_otras<br>Show the field ONLY if:<br>[po2_uds] = 9 | Otras Unidades | text<br>Custom alignment: RH |
| 343 | pco2_nr | pCO2 | radio<br>0 No Reallizado<br>1 Realizado<br>Custom alignment: RH |
| 344 | pco2<br>Show the field ONLY if:<br>[pco2_nr] = 1 | pCO2 | text (number)<br>Custom alignment: RH |

| | | | |
|---|---|---|---|
| 345 | pco2_uds<br>Show the field ONLY if:<br>[pco2_nr] = 1 | Unidades | radio<br>1 mmHg<br>2 kPa<br>9 Otras<br>Custom alignment: RH |
| 346 | pco2_uds_otras<br>Show the field ONLY if:<br>[pco2_uds] = 9 | Otras Unidades | text<br>Custom alignment: RH |
| 347 | hco3_nr | HCO3 | radio<br>0 No Reallizado<br>1 Realizado<br>Custom alignment: RH |
| 348 | hco3<br>Show the field ONLY if:<br>[hco3_nr] = 1 | HCO3 | text (number)<br>Custom alignment: RH |
| 349 | hco3_uds<br>Show the field ONLY if:<br>[hco3_nr] = 1 | Unidades | radio<br>1 mEq/L<br>2 mmol/L<br>9 Otras<br>Custom alignment: RH |
| 350 | hco3_uds_otras<br>Show the field ONLY if:<br>[hco3_uds] = 9 | Otras Unidades | text<br>Custom alignment: RH |
| 351 | lactato_nr | Lactato | radio<br>0 No Reallizado<br>1 Realizado<br>Custom alignment: RH |
| 352 | lactato<br>Show the field ONLY if:<br>[lactato_nr] = 1 | Lactato | text (number)<br>Custom alignment: RH |
| 353 | lactato_uds<br>Show the field ONLY if:<br>[lactato_nr] = 1 | Unidades | radio<br>1 g/dL<br>2 g/L<br>9 Otras<br>Custom alignment: RH |
| 354 | lactato_uds_otras<br>Show the field ONLY if:<br>[lactato_uds] = 9 | Otras Unidades | text<br>Custom alignment: RH |
| 355 | analitica_y_laboratorio_complete | Section Header: *Form Status*<br>Complete? | dropdown<br>0 Incomplete<br>1 Unverified<br>2 Complete |
| Instrument: **Datos Radiologicos** (datos_radiologicos) | | | |
| 356 | rx_torax | Radiografía de Tórax | radio<br>0 No<br>1 Si<br>Custom alignment: RH |
| 357 | rx_torax_fecha<br>Show the field ONLY if:<br>[rx_torax] = '1' | Fecha<br>*dd-mm-aaaa hh:mm* | text (datetime_dmy)<br>Custom alignment: RH |
| 358 | rx_torax_diagnostico<br>Show the field ONLY if:<br>[rx_torax] = '1' | Diagnóstico | text<br>Custom alignment: RH |
| 359 | rx_torax_imagen_patologica<br>Show the field ONLY if:<br>[rx_torax] = '1' | Imagen patologica | radio<br>0 No<br>1 Si<br>Custom alignment: RH |

| | | | |
|---|---|---|---|
| 360 | rx_torax_patron_intersticial<br>Show the field ONLY if:<br>[rx_torax] = '1' | Patron intersticial | radio<br>0 No<br>1 Si<br>Custom alignment: RH |
| 361 | rx_torax_neumonia<br>Show the field ONLY if:<br>[rx_torax] = '1' | Neumonia | radio<br>0 No<br>1 Si<br>Custom alignment: RH |
| 362 | rx_torax_neumonia_lateral<br>Show the field ONLY if:<br>[rx_torax_neumonia] = '1' | Neumonia | radio<br>1 Unilateral<br>2 Bilateral<br>Custom alignment: RH |
| 363 | rx_torax_lobulos_afectados<br>Show the field ONLY if:<br>[rx_torax_neumonia] = '1' | Lobulos afectados | checkbox<br>IS rx_torax_lobulos_afectados___is Izquierdo Superior<br>II rx_torax_lobulos_afectados___ii Izquierdo Inferior<br>DS rx_torax_lobulos_afectados___ds Derecho Superior<br>DM rx_torax_lobulos_afectados___dm Derecho Medio<br>DI rx_torax_lobulos_afectados___di Derecho Inferior<br>Custom alignment: LV |
| 364 | tac_torax | Tomografía Computarizada de Tórax | radio<br>0 No<br>1 Si<br>Custom alignment: RH |
| 365 | tac_torax_fecha<br>Show the field ONLY if:<br>[tac_torax] = '1' | Fecha<br>*dd-mm-aaaa hh:mm* | text (datetime_dmy)<br>Custom alignment: RH |
| 366 | tac_torax_diagnostico<br>Show the field ONLY if:<br>[tac_torax] = '1' | Diagnóstico | text<br>Custom alignment: RH |
| 367 | tac_torax_imagen_patologica<br>Show the field ONLY if:<br>[tac_torax] = '1' | Imagen patologica | radio<br>0 No<br>1 Si<br>Custom alignment: RH |
| 368 | tac_torax_patron_intersticial<br>Show the field ONLY if:<br>[tac_torax] = '1' | Patron intersticial | radio<br>0 No<br>1 Si<br>Custom alignment: RH |
| 369 | tac_torax_neumonia<br>Show the field ONLY if:<br>[tac_torax] = '1' | Neumonia | radio<br>0 No<br>1 Si<br>Custom alignment: RH |
| 370 | tac_torax_neumonia_lateral<br>Show the field ONLY if:<br>[tac_torax_neumonia] = '1' | Neumonia | radio<br>1 Unilateral<br>2 Bilateral<br>Custom alignment: RH |

| | | | |
|---|---|---|---|
| 371 | tac_torax_lobulos_afectados<br>Show the field ONLY if:<br>[tac_torax_neumonia] = '1' | Lobulos afectados | checkbox<br>IS \| tac_torax_lobulos_afectados___is \| Izquierdo Superior<br>II \| tac_torax_lobulos_afectados___ii \| Izquierdo Inferior<br>DS \| tac_torax_lobulos_afectados___ds \| Derecho Superior<br>DM \| tac_torax_lobulos_afectados___dm \| Derecho Medio<br>DI \| tac_torax_lobulos_afectados___di \| Derecho Inferior<br>Custom alignment: LV |
| 372 | datos_radiologicos_complete | Section Header: *Form Status*<br>Complete? | dropdown<br>0 \| Incomplete<br>1 \| Unverified<br>2 \| Complete |
